# Hepatitis-B immune status and vaccination in Family Medicine Practice: A retrospective study

**DOI:** 10.12669/pjms.40.6.8371

**Published:** 2024-07

**Authors:** Olgun Goktas

**Affiliations:** 1 Olgun Goktas Associate Professor, Uludag University Family Health Center, Nilufer, Bursa, Turkey

**Keywords:** Hepatitis-B, Vaccination, Immunity, Healthcare Workers, Family Medicine Practice

## Abstract

**Objective:**

To examine the relationship between Hepatitis B vaccination status and demographic and hepatitis B markers of individuals receiving healthcare services.

**Methods:**

The study designed in a retrospective structure using quantitative research methods was conducted with a total of 1837 individuals including medical school students and other healthcare professionals (975 female, 862 male) aged 19-77 who were registered to the Family Health Center between March 1, 2023, and March 31, 2023. In the study, sociodemographic characteristics, infancy and adult full-dose hepatitis-B vaccines, and marker laboratory measurements of these individuals who were registered to the Family Health Center were examined. SPSS 25.00 program was used for statistical analysis and the level of significance was determined as 0.05.

**Results:**

In general, the rate of three doses of vaccination in infancy was 55.1%. Vaccine doses administered to individuals were one with 15.1%, two with 22.9%, and three with 62.1%. In the study, it was determined that anti-HBs levels before and after vaccination differed significantly according to vaccine doses (p=0.01). It was determined that the anti-HBs levels of the three doses vaccine group were 100 and above (p=0.01).

**Conclusion:**

Although the Universal Hepatitis-B Vaccination Program was followed in our study, it was determined that antibody levels in healthcare workers decreased or ended over time, and hepatitis-B antibody levels increased significantly with each dose of vaccine administered. For this reason, it is of great importance to determine regular antibody levels and develop standard vaccination programs, especially in healthcare workers.

## INTRODUCTION

Hepatitis-B virus infection poses a significant risk for the normal population and especially for healthcare workers. Hepatitis-B vaccination is effective and provides safe protection against infection. The World Health Organization (WHO) reports that vaccination is the most effective way to combat hepatitis-B infection worldwide. As stated in the Universal Hepatitis-B Vaccination Program launched in 1997, it recommends that all babies be vaccinated at 0, 1, and six months from birth. It also suggested that target and risk groups should be determined and countries should act programmatically in this regard within their health policies. It also submits its updated opinions to provide guidance to member states. These recommendations are in line with the use of vaccines in large-scale immunization programs.^1^,^2^

In a study investigating the global barriers to hepatitis-B vaccination, it is stated that the reasons such as the inability to detect especially unvaccinated children, lack of awareness and education in individuals, poor communication of healthcare professionals, problems with beneficiaries and service providers, and inadequate access are important. The study highlights the limited availability and accessibility of healthcare provider-based immunization and indicates that barriers vary from country to country. Emphasis is placed on issues such as insufficient knowledge of healthcare professionals in dosing and inadequate screening of high-risk groups.^3^ It is reported that the records of vaccination against hepatitis-B and serological status research among community health workers are insufficient and incomplete in most people. Although most workers were vaccinated and demonstrated an immunological response to hepatitis-B, susceptibility was found after one dose of vaccination (threat dose).^4^

There are differences in the literature on hepatitis-B immunity status and vaccinations.^5^-^7^ In terms of immunization, extensive and detailed studies are needed due to vaccine procedures, administered doses, time differences, and application inadequacies.^8^-^10^ In this study, we aimed to examine the relationship between Hepatitis B vaccination status and demographic and hepatitis B marker indicators of individuals receiving health care in family medicine between 2010 and 2022. The research question in the study was determined as “Do individuals’ vaccine doses differ according to demographic characteristics and hepatitis B marker results?”

## METHODS

In the study designed in a retrospective structure using quantitative research methods, quantitative comparative research methods were used to test the hypotheses. Patient records, which are secondary data sources, were used as a data collection method. In the study, all data belonging to these individuals who were registered to the Family Health Center between March 1, 2023, and March 31, 2023, were retrieved from the database (Family Medicine Information Registration System) retrospectively. In the study, it was determined that the total number of patients over the age of 18 registered in the Family Health Center region between 1-31 March 2023 was 4345 (N). Simple random sampling method determined that at least 1064 patients should participate in the study with 1% error and 95% confidence. The study was conducted with 1837 patients with valid data. Sociodemographic characteristics, hepatitis, current or previous liver disease history, infancy, and adult full dose vaccinations, hepatitis-B marker laboratory measurements, all hepatitis-B vaccines, and laboratory results of hepatitis-B markers made for control after vaccination were determined and recorded. Hepatitis-B vaccination status, hepatitis-B marker (Chemiluminescent enzyme immunoassay-CLIA for hepatitis B surface antigen (HBsAg) and microparticle enzyme immunoassay-MEIA for antibody to hepatitis B surface antigen (anti-HBs)) levels and the relationships between them were examined. While the significance level was determined as 0.05, the analyzes were made with the SPSS 25.00 program.

### Ethical approval

This retrospective study was performed after receiving the approval of the Clinical Research Ethics Committee of Bursa Uludag University (Reference no: 2023-7/16, dated: 11.04.2023) and in accordance with the Declaration of Helsinki.

### Statistical analysis

In the study, general features are given as frequency and percentage. The chi-square test was used to examine the demographic and vaccine-related protection levels according to the doses of the participants. Cramer’s v test was used to examine the differences in pre-vaccine antibody and antigen levels according to vaccine groups. In the study, p<0.05 was accepted as the critical decision value. The data were analyzed using the SPSS 25.0 (Statistical Packages of Social Sciences) program on the computer.

## RESULTS

In our study, 1374 (74,82%) of the 1837 participants aged between 19 and 77 were medical faculty students and 463 (25.2%) were health workers. It was determined that the rate of three doses of vaccination in infancy recommended by the World Health Organization (WHO) was 55.1%.

In general, pre-vaccination antigen (HBsAg) levels of individuals were one and above with a rate of 0.2%. Pre-vaccination antibody (anti-HBs) levels were found to be 10 and below with a rate of 53.1%, between 10-100 with a rate of 13.6%, and 100 and above with a rate of 33.3%.

In general, vaccine doses administered to individuals were 1 with a rate of 15.1%, two with a rate of 22.9%, and three with a rate of 62.1%. After vaccination, control antibody (anti-HBs) levels were found to be 10 and below with a rate of 0.3%, between 10-100 with a rate of 1.3%, and 100 and above with a rate of 98.4%, (Table-I).

**Table-I:** General characteristics.

		n	%
Sex	Male	862	46.9%
	Female	975	53.1%
Group	Medical faculty student	1374	74.8%
	Healthcare professionals	463	25.2%
History of hepatitis or liver disease	No	1830	99.8%
	Yes	3	0.2%
3 Doses of primary vaccination in infancy	No	807	44.9%
	Yes	992	55.1%
Pre-assay vaccination status	No	949	99.0%
	Yes	10	1.0%
Antigen (HBsAg) level before vaccination	0-0.99	935	99.8%
	1 and above	2	0.2%
Antibody (anti-HBs) level before vaccination	<10	497	53.1%
	10-100	127	13.6%
	>100	312	33.3%
Number of vaccine doses. if administered	1	116	15.1%
	2	176	22.9%
	3	478	62.1%
Control HBsAg level after vaccination	0-0.99	631	99.8%
	1 and above	1	0.2%
Control anti-HBs level after vaccination	<10	2	0.3%
	10-100	8	1.3%
	>100	622	98.4%

Hepatitis-B Surface Antigen (HBsAg) level (Result unit; S/CO. reference; <1) Hepatitis-B Surface Antibody (anti-HBs) level (Result unit; mIU/mL. reference; <10).

It has been determined that vaccine doses differ according to gender. The reason for the difference was that the second and third dose rates of female participants were significantly higher than men (p=0.01). It was determined that the 1st, 2nd, and 3rd dose vaccine rates were not significantly different in the group of medical students or healthcare professionals (p=0.42).

It was also observed that the antibody (anti-HBs) rates before vaccination were different. The difference was found to be between 10-100 with a higher rate of anti-HBs levels in the group that received a single-dose vaccine (p=0.04). Post-vaccination antibody (anti-HBs) rates were different according to dose. The difference was found to be between 100 and above with a higher rate of anti-HBs levels in the group that received two and three doses of vaccine (p=0.04). In addition, it was observed that the anti-HBs levels were between 10-100 with a higher rate in the single-dose group, Table-II).

**Table-II:** General characteristics by vaccine dose.

		Vaccine dose			p

		1	2	3	

		n	n	n	
Sex	Male	58 (50.0%)	65 (36.9%)	220 (46.0%)	0.01*
	Female	58 (50.0%)	111 (63.1%)	258 (54.0%)	
Group	Medical faculty student	100 (86.2%)	147 (83.5%)	402 (84.1%)	0.42
	Healthcare professionals	16 (13.8%)	29 (16.5%)	76 (15.9%)	
3 Doses of primary vaccination in infancy	No	38 (33.6%)	81 (46.6%)	187 (40.0%)	0.15
	Yes	75 (66.4%)	93 (53.4%)	280 (60.0%)	
Pre-assay vaccination status	No	84 (96.6%)	144 (100.0%)	312(100.0%)	0.96
	Yes	3 (3.4%)	0 (0%)	0 (0%)	
	1 and above	0 (0%)	0 (0%)	0 (0%)	
Antibody (anti-HBs) level before vaccination	<10	65 (82.3%)	127 (92.0%)	270 (91.5%)	0.04*
	10-100	12 (15.26%)	11(8.0%)	25 (8.5%)	
	>100	2 (2.5%)	0 (0%)	0 (0%)	
Post-vaccine Control (anti-HBs) level	<10	2 (5.6%)	0 (0%)	0 (0%)	0.04*
	10-100	4 (11.1%)	2 (1.4%)	2 (0.4%)	
	>100	30 (83.3%)	138 (98.6%)	454 (99.6%)	

**Chi-square analysis.

* Significant correlation at the 0.05 level Hepatitis-B Surface Antigen (HBsAg) level (Result unit; S/CO. reference; <1) Hepatitis-B Surface Antibody (anti-HBs) level (Result unit; mIU/mL. reference; <10).

In the study, it was determined that HBsAg levels before and after vaccination differed significantly according to vaccine doses (p=0.01). The reason for the difference was that the HBsAg levels of the participants who received one dose before the vaccine were at a higher rate of 0-0.99, while the HBsAg levels of the 3rd dose group were higher than one and above after the vaccine, (Table-III).

**Table-III:** HBsAg Before and after vaccination.

			Vaccine dose			p

			1	2	3	
Antigen (HBsAg) level before vaccination	0-0.99	n	79	138	295	0.01*
		%	15.4%	27.0%	57.6%	
	1 and above	n	0	0	0	
		%	0.0%	0.0%	0.0%	
Post-vaccine control antigen (HBsAg) level	0-0.99	n	36	139	456	
		%	5.7%	22.0%	72.3%	
	1 and above	n	0	1	0	
		%	0.0%	100.0%	0.0%	

**Caramer V test.

* Significant difference at 0.05 level Hepatitis-B Surface Antigen (HBsAg) level (Result unit; S/CO. reference; <1) Hepatitis-B Surface Antibody (anti-HBs) level (Result unit; mIU/mL. reference; <10).

In the study, it was determined that anti-HBs levels before and after vaccination differed significantly according to vaccine doses (p=0.01). The reason for the difference was that while the anti-HBs levels of the participants who had 2-3 doses before the vaccine were lower than 10 at a higher rate, these levels were mostly 10 and above after the vaccine. It was observed that the anti-HBs levels of the 3rd dose group were 100 and above, (Table-IV).

**Table-IV:** Anti-HBs before and after vaccination.

			Vaccine dose			p

			1	2	3	
Antibody (anti-HBs) level before vaccination	<10	n	65	127	270	0.01*
		%	14.1%	27.5%	58.4%	
	10-100	n	12	11	25	
		%	25.0%	22.9%	52.1%	
	>100	n	2	0	0	
		%	100.0%	0.0%	0.0%	
Post-vaccine control antibody (anti-HBs) level	<10	n	2	0	0	
		%	100.0%	0.0%	0.0%	
	10-100	n	4	2	2	
		%	50.0%	25.0%	25.0%	
	>100	n	30	138	454	
		%	4.8%	22.2%	73.0%	

**Caramer V test.

* Significant difference at 0.05 level Hepatitis-B Surface Antigen (HBsAg) level (Result unit; S/CO. reference; <1) Hepatitis-B Surface Antibody (anti-HBs) level (Result unit; mIU/mL. reference; <10)

## DISCUSSION

In our study, it was determined that the rate of three doses of vaccination in infancy recommended by the World Health Organization (WHO) was 55.1%. This rate is probably composed of medical faculty students in the younger age group. It is evident that older healthcare workers are not vaccinated at the full dose in infancy.

In general, antigen (HBsAg) and antibody (anti-HBs) levels in laboratory tests performed before vaccination were not at the desired level, especially in medical school students and other health personnel. It is clear that this situation necessitates the need for vaccination. For this reason, the generally administered vaccines of these individuals were examined. The administered vaccine doses were one with a rate of 15.1%, two with a rate of 22.9%, and three doses with a rate of 62.1%. Control antibody (anti-HBs) levels were found to be 10 and below with 0.3%, between 10-100 with a rate of 1.3%, and 100 and above with a rate of 98.4% after the vaccines were administered in parallel with these dose numbers. These results showed that the vaccine provides a protective immunity level even with a single dose, but it provides very high levels of protection with three doses. These protective levels are important for various healthcare professionals such as medical school students, nurses, doctors, and health technicians.

In a study, it is suggested that three doses of vaccination, although with different dose ranges, provide very high protective antibody levels in healthcare workers and that this vaccine should be administered to healthcare workers.^11^ On the other hand, the risk of hepatitis-B and C disease is high in the use of unsterilized therapeutic materials. For this reason, it is important to raise awareness and vaccination of some occupational groups other than health personnel.^12^,^13^

It is stated that at least one dose of hepatitis-B vaccine is mandatory for health workers due to the increase in the rate of the vulnerable 5-10 years after vaccination.^14^ In a study in which it was stated that full dose vaccination was insufficient in university students, it is recommended that vaccination be mandatory before clinical practice internship.^15^

In another study, it was reported that 47.9% of children had protective anti-HBs antibody levels 10 years after primary vaccination in infancy.^16^ In this study, up to 50% protection achieved over a 10-year period is acceptable for the normal population. However, it should be aimed that this ratio is close to full in healthcare professionals and medical school students. Similarly, in our study, it was found that 58.4% of the vaccinated had a protective level approximately 20 years after the primary vaccination. At the end of the regular control and vaccination program of medical students and health workers in our family health center, it was determined that protective levels of up to 100% were achieved in all of them.

Protection with hepatitis-B vaccine is important not only for health personnel, but also for individuals who will be operated^17^ and at risk for chronic diseases.^18^,^19^ In one study, it is suggested that anti-HBs levels decrease after 18 years and that at least one dose of booster vaccine should be given.^20^ Especially medical school students, other health workers, and risky groups should be protected and followed up with a systematic and standard vaccination program.^21^ The contribution of the results of our study, which evaluates the data of individuals in this group in family medicine, to the literature is important. A systematic review and meta-analysis study showed that the vaccine protocol applied was effective in protecting healthcare workers.^22^

### Limitations

Other infectious conditions affecting healthcare workers, such as Hepatitis-A and hepatitis C, were not evaluated in the study.

## CONCLUSION

Although the Universal Hepatitis-B Vaccination Program was followed in our study, it was determined that antibody levels in healthcare workers decreased or ended over time, and hepatitis-B antibody levels increased significantly with each dose of vaccine administered. For this reason, it is of great importance to determine regular antibody levels and develop standard vaccination programs, especially in healthcare workers.

### Authors’ Contribution:

**OG:** Conceived, designed, data collection, statistical analysis & editing of the manuscript, manuscript writing, did review and final approval of the manuscript
